# Inhibition of IAPP aggregation by insulin depends on the insulin oligomeric state regulated by zinc ion concentration

**DOI:** 10.1038/srep08240

**Published:** 2015-02-04

**Authors:** Praveen Nedumpully-Govindan, Feng Ding

**Affiliations:** 1 Department of Physics and Astronomy, Clemson University, Clemson, SC 29634, USA

## Abstract

While islet amyloid polypeptide (IAPP) aggregation is associated with β-cell death in type-II diabetes (T2D), environmental elements of β-cell granules — e.g. high concentrations of insulin and Zn^2+^ — inhibit IAPP aggregation in healthy individuals. The inhibition by insulin is experimentally known, but the role of Zn^2+^ is controversial as both correlations and anti-correlations at the population level are observed between T2D risk and the activity of a β-cell specific zinc ion transporter, ZnT8. Since Zn^2+^ concentration determines insulin oligomer equilibrium, we computationally investigated interactions of IAPP with different insulin oligomers and compared with IAPP homodimer formation. We found that IAPP binding with insulin oligomers competes with the formation of both higher-molecular-weight insulin oligomers and IAPP homodimers. Therefore, zinc deficiency due to loss-of-function ZnT8 mutations shifts insulin oligomer equilibrium toward zinc-free monomers and dimers, which bind IAPP monomers more efficiently compared to zinc-bound hexamers. The hetero-molecular complex formation prevents IAPP from self-association and subsequent aggregation, reducing T2D risk.

Diabetes mellitus is a metabolic disease affecting an estimated 383 million people worldwide, of which about 90% suffer from T2D. T2D features an adult onset of the disease and its progression is characterized by pancreatic β-cell death, causing reduced insulin secretion. The disease mechanism of T2D is largely unknown, and there is no known cure. Given the complex nature of the disease, the cause of β-cell death is likely the result of interplay of many factors. For instance, since amyloid aggregates of IAPP (a.k.a. amylin) in pancreas are found in approximately 90% of patients upon postmortem examination, many research efforts focused on understanding IAPP aggregation and its association with the disease^1^,^2^,^3^,^4^. Other disease-related molecules have also been pursued, e.g. genome-wide association studies (GWAS) to find genetic variations resulting in an increased or decreased T2D risk^5^,^6^. Uncovering the inter-connection of various disease-related factors is crucial for our understanding of the diseases and can help identify potential targets to develop therapeutics against the disease.

IAPP is a 37-residue peptide secreted by pancreatic β-cells. While it is under debate whether the aggregation of IAPP is the cause or merely the consequence of β-cell death, accumulating evidences suggest that IAPP aggregates — either insoluble amyloid or soluble oligomers — are toxic to β-cells^7^,^8^,^9^. For example, IAPP variants of diabetes-prone cat and human aggregate readily *in vitro*, while IAPP peptides of diabetes-free rat and pig have low propensity to form amyloid aggregates^10^. Although these IAPP variants differ only in a few amino acids, many of these mutations significantly alter the aggregation propensity^8^,^11^. For example, a naturally occurring polymorphic Ser20Gly mutation renders the human IAPP more aggregation-prone^8^,^12^, and an Asian subpopulation carrying this mutation is subjected to the early onset of T2D. Moreover, transgenic mice expressing human IAPP develop hyperglycemia and diabetes. Therefore, aggregation of IAPP plays an important role in T2D. *In vitro* studies revealed that human IAPP aggregates readily at *μM* concentrations^13^. However, the peptide is stored in β-cell granules at *mM* concentrations without apparent formation of amyloid aggregations in healthy individuals^14^. Hence, environmental elements of β-cell granules — e.g. low pH^15^, high concentrations of zinc ion and insulin peptides — inhibit the formation of IAPP aggregates. The presence of a high concentration of insulin, usually 20–100 times higher molar fraction compared to IAPP^16^, is believed to be responsible for the prevention of IAPP aggregation. *In vitro* studies show that insulin, indeed, inhibits or slows down IAPP aggregation^17^,^18^. Despite many research efforts^17^,^18^,^19^,^20^,^21^, the detailed mechanism of the inhibition of IAPP aggregation by insulin remains unknown.

In addition to IAPP and insulin with their important roles in T2D, genetics association studies have identified other T2D-related genes and corresponding variations across different populations with distinct diabetes risks. For example, an earlier genome-wise association study (GWAS)^5^ identified about a dozen genes associated with increased T2D risks. Of particular interest among these identified genes is the variation of gene SLC30A8, which encodes a zinc transporter, ZnT8, specific to β-cells. ZnT8 transports Zn^2+^ ions against the concentration gradient into β-cell granules^22^. A high concentration of zinc ions is important for the formation of insulin hexamer, which is stored in the crystal form in the β-cell granules. It was found that an activity-reducing Trp325Arg mutation of ZnT8^23^ results in a greater T2D risk^5^. However, follow-up studies with ZnT8 knockout mice were inconclusive about the correlation between decreased zinc concentration^24^ and the development of diabetic conditions^23^,^24^,^25^. Interestingly, a recent GWAS study reports a “seemingly opposite” effect: loss-of-function mutations of SLC30A8 protects against diabetes with a high statistical significance^6^. A fundamental question is what the effect of zinc-deficiency in β-cell granules is on the disease development, such as IAPP aggregation and IAPP-insulin interactions.

Insulin can exist as monomers, dimers or hexamers^26^. Zinc ion binds only to hexamers, which are usually insoluble and from crystals in the granule. Therefore, the concentration of Zn^2+^ determines the equilibrium of insulin oligomers. We hypothesize that the inhibition of IAPP aggregation by insulin depends on insulin oligomeric states, which is regulated by zinc concentration in the β-cell granule. We plan to perform computer simulations to understand the structural and thermodynamic features of IAPP and its interactions with different insulin oligomers. Previous computational studies focused on understanding the structure and dynamics of IAPP monomers and dimers^27^,^28^,^29^,^30^, and the dependence of IAPP aggregation on sequence variations^31^. A few experimental and computational studies of IAPP-insulin interactions have been reported^17^,^18^,^19^,^20^,^21^,^32^; however, the dependence on insulin oligomeric states remained to be investigated and a partial IAPP peptide was used in the computational study^21^. In this work, we perform molecular dynamics (MD) simulations to study the binding of hIAPP with soluble insulin monomers and dimers. Because of the large system size with multiple proteins interacting with each other and the requirement of long time scale simulations necessary to reach equilibrium, systematic study using traditional MD simulations is computationally challenging and resource-demanding. Instead, we use the atomistic discrete molecular dynamics (DMD) simulations with implicit solvent to model the molecular system^33^. DMD is a special type of MD algorithm, featuring accurate modeling and rapid sampling efficiency of protein conformational dynamics^34^,^35^ and protein-protein interactions^36^,^37^. Using DMD simulations, we find that IAPP binds insulin monomers and dimers preferentially at specific surface regions that are buried in the zinc-bound insulin hexamer. The regions of IAPP that bind insulin oligomers are also responsible for the IAPP homodimer formation. Therefore, our computational study provides a molecular insight to the recent GWAS findings^6^ in that loss-of-function of ZnT8 and subsequent Zn^2+^ deficiency in β-cell granules shift the insulin oligomer equilibrium toward insulin monomers and dimers, which bind IAPP monomers efficiently and prevent IAPP from self-association and aggregation, thus reducing T2D risk.

## Results

We first performed DMD simulations of human and rat IAPP (hIAPP and rIAPP, respectively) to investigate the possible effect of sequence variations on the structural and thermodynamic properties of IAPP monomers. We further studied the formation of hIAPP homodimer, and molecular complex foramation between a hIAPP and an insulin monomer or dimer. The details of molecular systems used in DMD simulations can be found in the Methods section.

### The hIAPP monomer does not have a well-defined folded state

**Figure 1:**
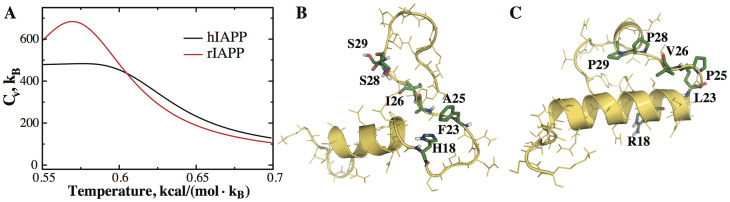
hIAPP and rIAPP monomer structures. (A) The specific heat, *C_v_*, plots for hIAPP and rIAPP monomers. The hIAPP monomer lacks any peak in the *C_v_* plot while the rIAPP monomer has a well-defined melting temperature. Representative snapshots of hIAPP (B) and rIAPP (C) conformations are taken from DMD simulations at T = 0.55 kcal/(mol·K_B_). Compared to hIAPP, rIAPP has an ordered 3D folded structure. Six residues different between hIAPP and rIAPP are highlighted in stick representation.

With the sequence difference of only 6 residues, hIAPP aggregates rapidly *in vitro* while rIAPP does not. To uncover the molecular mechanism behind this striking difference, we first performed replica exchange DMD (REXDMD) simulations of hIAPP and rIAPP monomers to study their folding thermodynamics in solution (Methods). NMR structures solved in the hydrophobic environment were used as the starting conformations^38^. From the replica exchange simulations, we computed the temperature dependence of specific heat and the secondary structure contents using weighted histogram analysis method (WHAM)^39^. Within the temperature range of our simulations, the specific heat of hIAPP decreases slowly and steadily with increasing temperatures, lacking any peak (Fig. 1A). In contrast, a distinct peak exists in the specific heat curve of rIAPP around T = 0.575 kcal/mol·*K_B_* (see Methods for DMD units; Kelvin or °C units are not used to indicate that temperature values in DMD simulation do not directly correspond to actual temperatures), which indicates a more corporative unfolding transition compared to hIAPP. The secondary structure contents of hIAPP and rIAPP exhibit similar behaviors. Both peptides are helical at low temperature. As temperature increases, the helical content of hIAPP decreases and coil content increases gradually (Fig. S1). In the case of rIAPP, the helical content exhibits a sharp decrease around the same transition temperature of 0.575 kcal/mol·*K_B_* (~290 K) and the random coil structure content of rIAPP increases significantly around the same temperature. This observation is consistent with a previous NMR study of rIAPP at the room temperature (~300 K), where an extended structural ensemble of rIAPP with residual helical content was reported^40^.

We also examined the three-dimensional structures of both peptides at different temperatures. At a low temperature T = 0.5 kcal/mol·*K_B_*, the N-terminal residues 5–17 and central residues 20–25 of hIAPP form two helices. As the temperature increases, the helix formed by residues 5–17 remains stable while residues 20–25 of hIAPP lose their helical structure first (e.g. a typical structure at T = 0.55 kcal/mol·*K_B_* in Fig. 1B). In contrast, rIAPP residues 5–23 form a single helix at T = 0.5 kcal/mol·*K_B_* with the C-terminal packing against the N-terminal helix (Fig. 1C). The structure remains intact at 0.55 kcal/mol·*K_B_*, and unfolds above its melting temperature (Fig. 1A). Comparing the structures of hIAPP and rIAPP, we found that mutations of six residues in rIAPP with respect to hIAPP (highlighted in sticks in Figs. 1B&C) are responsible for tertiary contacts formation. Therefore, our simulations suggest that in contrast to its rat variant hIAPP monomer does not have a well-defined folded state and the lack of tertiary structures may promote aggregation.

### The hIAPP dimer is more ordered than monomer

**Figure 2:**
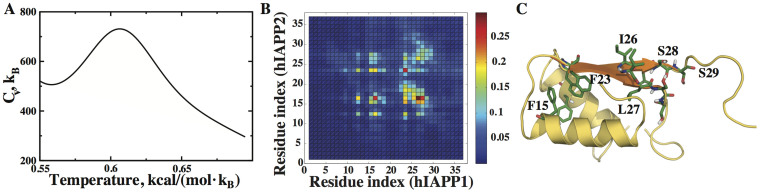
hIAPP homodimer formation. (A) The specific heat plot of hIAPP dimer has a peak, which is different from its monomer. (B) Inter-molecular contact frequencies of hIAPP residues when they form dimers at T = 0.6 kcal/(mol·K_B_). Many of the frequent contacts are along the diagonal, indicating a favorable parallel association of two monomers. (C) A typical dimer structure corresponding to the centroid of one of the largest clusters shows a parallel association of helices formed by N-terminal residues. Residues 22–29 in orange, which are amyloidogenic, form a β-sheet in the dimer. The hotspot residues making frequent contacts are highlighted in stick representation.

Dimerization is likely the first step in hIAPP aggregation pathway^41^. We first performed replica exchange simulations of hIAPP dimer with two monomers initially positioned in proximity with a parallel orientation (Methods). We computed the specific heat as the function of temperature. The specific heat plot featured a distinct peak (Fig. 2A), which suggests that dimerization has a stabilizing effect on hIAPP structure and the peak corresponds to dimer dissociation. In order to characterize structural properties of hIAPP dimers, we carried out equilibrium DMD simulations at T = 0.6 kcal/mol·*K_B_* (~300 K), which is below the dimer dissociation temperature. Multiple independent simulations with different initial velocities and relative dimer orientations were carried out in order to increase the statistical significances of simulation results. With these constant temperature DMD simulations, we computed the inter-monomer contact frequency for all residue pairs. Two residues are defined to form a contact if any two inter-residue heavy atoms are within 5 Å. Interestingly, we found a few hotspots in the contact frequency map (Fig. 2B). Many of the frequent contacts are along the diagonal, suggesting a parallel association between the two monomers. In particular, residues with strong inter-monomer interactions include Leu12, Phe15, Leu16, Phe23, Ile26, Leu27 and Ser28. Three of the most frequently contacting residues (Phe23, Ile26 and Ser28) are among the six mutations between hIAPP and rIAPP variants, suggesting their important roles in hIAPP dimerization. For instance, a point mutation of Ile26 to proline was shown to make the mutant peptide not only non-aggregating but also an inhibitor of the wild-type hIAPP aggregation^11^. Mutations of Phe15 was also found to significantly affect hIAPP aggregation in experiments^41^.

To further verify whether hIAPP monomers actually form parallel dimer structures, we clustered low energy structures obtained from DMD simulations using a hierarchal clustering algorithm^42^ (Methods) and examined the centroid representatives of the most populated clusters (Fig. S2). We found that the highly populated clusters indeed consist of parallel β-sheet structures formed by residues between 20–30. The N-termini are mostly helical, which can also make parallel contacts with each other (Fig 2C). Wiltzius *et al*. solved the structure of hIAPP fused to a maltose binding protein in the dimeric form^41^. They reported parallel association of N-terminal helices with aromatic stacking between Phe15 residues of two hIAPP monomers, in agreement with some of our DMD-derived structural ensembles (Fig. 2C). Interestingly, the amyloid-predicting algorithm, Waltz^43^, also predicted that residues 22–29 of hIAPP are amyloidogenic (colored orange in Fig. 2C) whereas rIAPP is not amyloidogenic. Therefore, the self-association tendency of these residues in DMD simulations is consistent with the amyloidogenic nature of the same region. Our DMD simulations support the hypothesis that dimerization is the first step along the hIAPP aggregation pathway^41^.

### The binding of hIAPP with insulin monomers and dimers competes with high-order insulin oligomer formation and hIAPP dimerization

**Figure 3:**
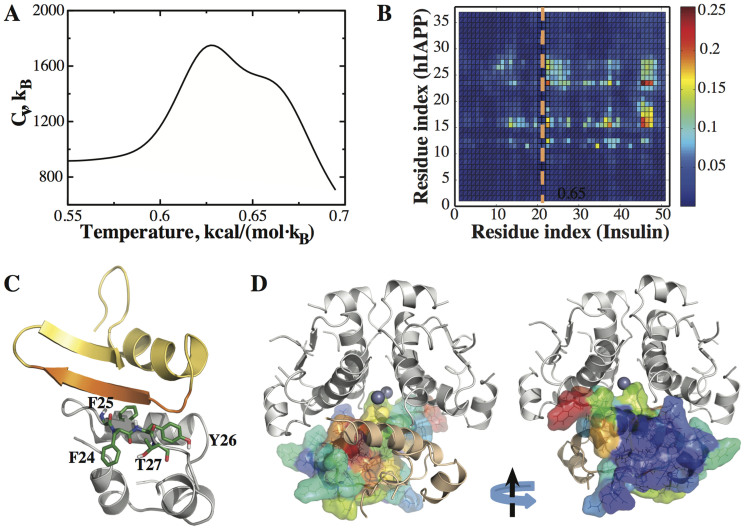
hIAPP–insulin dimerization. (A) The specific heat plot for insulin–hIAPP complex has two peaks corresponding to insulin–hIAPP unbinding and insulin melting. (B) The inter-molecular contact frequencies between insulin and hIAPP residues at T = 0.6 kcal/(mol·K_B_). Insulin A-chain (21 residues) and B-chain (30 residues), separated by a dotted orange line, are numbered sequentially. (C) A typical structure of the hIAPP-insulin complex is derived from DMD simulations. The amyloidogenic residues of hIAPP (residues 22–29) are shown in orange. The residues of insulin important for binding hIAPP are highlighted in stick representation. (D) The residues of an insulin monomer are colored according to hIAPP binding frequencies in the structure of an insulin hexamer. The view with 180° rotation is also presented. The residues with strong hIAPP-binding are located at the insulin monomer–monomer interface.

We carried out both replica exchange and constant temperature DMD simulations of a hIAPP monomer together with a human insulin “monomer”, which consists of two chains (A- and B-chain consisting of 21 and 30 residues, respectively) cross-linked by a disulfide bond. We observed two peaks in the specific heat plot (Fig. 3A), which may correspond to either the melting of individual molecules or their disassociation. To delineate these peaks, we calculated the number of inter-molecular contacts between insulin and hIAPP and the backbone Cα root-mean-square deviation (RMSD) of insulin as a function of temperature. We found that the first and second peaks in the specific heat plot (Fig. 3A) correspond to insulin–hIAPP dissociation and insulin unfolding, respectively (data not shown). The lack of peak corresponding to hIAPP unfolding is consistent with the non-cooperative unfolding of hIAPP monomer (Fig. 1A). Compared to the hIAPP dimer dissociation temperature (Fig. 2A), the hIAPP-insulin dissociation temperature is higher, suggesting a stronger inter-molecular interaction. The binding of hIAPP with insulin monomer also considerably reinforces the helical content of hIAPP as the melting of helices happens at a higher temperature (Fig. S3) comparing to the monomer (Fig. S1).

Based on independent constant temperature DMD simulations at T = 0.6 kcal/mol·*K_B_*, we calculated inter-molecular contact frequencies (Fig. 3B). The hIAPP residues that make frequent contacts with insulin mostly include 11–16 and 23–27 (y-axis in Fig. 3B). Most of these residues are the exact same residues making inter-monomer contacts in the hIAPP homodimer simulations (Fig. 2). For insulin, there are a few hotspots, including residues 44–48 near the C-terminal (i.e., residues 23–27 of B-chain as observed in a recent work^32^; Fig. 3C). To visualize the binding of insulin monomer with hIAPP, we colored insulin residues according to their hIAPP-binding frequencies (Fig. 3D). The surface area of insulin monomer with the most frequent binding to hIAPP coincides with the insulin monomer–monomer contact surface (and also to insulin–insulin dimer interface). A similar hIAPP-binding interface for insulin monomer has been observed in a computational study using a hIAPP segment (residues 9 to 20) with the amyloidogenic residues truncated^21^. We also examined the structures of the hetero-molecular complex using clustering analysis (Fig. S4). The representative structure of a large cluster consisting of ~11.6% of the total population (Fig. 3C) showed that the insulin B-chain residues 24–27 bind to the amyloidogenic region of hIAPP (residues 22–29), forming an antiparallel sheet. The C-terminal of hIAPP also contributes to the sheet formation, forming a third strand. In three out of four most populated clusters (account for ~43.6% of all conformations) the C-terminal residues and residues 22–29 form β-hairpins. Therefore, hIAPP-insulin monomer binding competes with the oligomerization of insulin dimer and hexamer as well as hIAPP dimerization.

**Figure 4:**
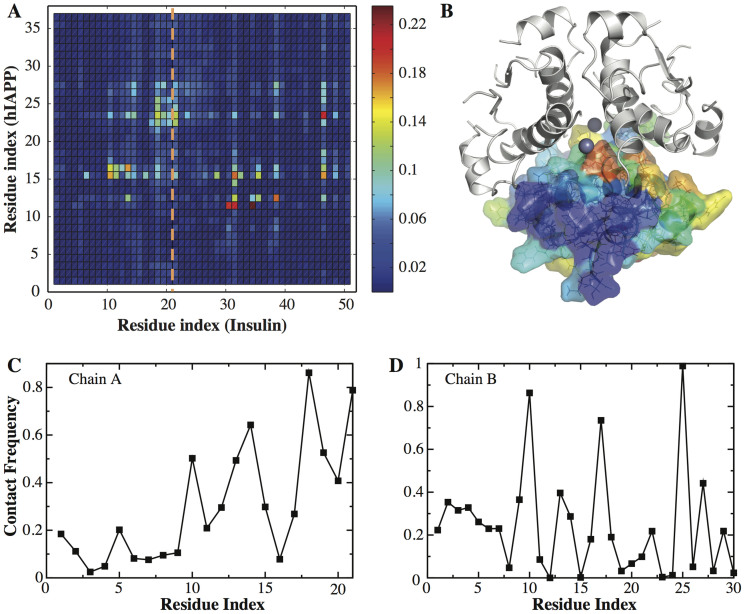
Insulin dimer–hIAPP contact. (A) The inter-molecular contact frequency map between insulin dimer (averaged over two monomers) and hIAPP. (B) The residues of an insulin dimer are colored according to the hIAPP-binding frequency in the 3D structure of the insulin hexamer. For each residue in A-chain (C) and B-chain (D), the average number of contacts with hIAPP is obtained from DMD simulations.

Next, we carried out constant temperature DMD simulations of an insulin dimer with a hIAPP monomer at the same temperature, T = 0.6 kcal/mol·*K_B_*. In a related study, the binding of rIAPP with insulin was probed by NMR chemical shift perturbation^20^. Since the concentration of insulin used in NMR study (0.5 *mM*) is higher than the insulin dimerization equilibrium constant (<100 *μM*), insulin are expected to be in its dimeric form and we compare the results with our hIAPP-insulin dimer simulations instead of the hIAPP-insulin monomer simulations. We computed the contact frequency map for insulin and hIAPP residues (Fig. 4A). We found a qualitative agreement between our simulation results and the chemical shift perturbation data in terms of the locations of peaks even though some of the details do not match perfectly. In NMR studies, chemical shift perturbation occurs primarily to the C-terminal half of A-chain and to the central residues of B-chain. Our contact frequency map follows this general trend (Fig. 4C and 4D). One major exception is the presence of a peak for the residue 46 (residue 25 of B-chain) in Fig. 4C which is not seen in the NMR studies. The difference might result from the fact that different IAPP sequences are used between experiments and simulations. We also color-coded insulin residues according to their binding frequencies to hIAPP (Fig. 4B). Interestingly, when the monomer–monomer interface of insulin is not available in the insulin dimer, hIAPP binds to insulin residues at the dimer–dimer interface. Insulin residues, which do not belong to either monomer–monomer or dimer-dimer interfaces and are solvent-exposed, also have weak probability to bind hIAPP monomer (Fig. 4B). Therefore, similar to hIAPP-insulin monomer binding, the association of hIAPP with insulin dimer also competes with the formation of hIAPP homodimer and high-molecular-weight insulin hexamer.

## Discussion

Using DMD simulations, we found structural differences between aggregation-prone hIAPP and non-aggregating rIAPP — hIAPP is intrinsically disordered while rIAPP can form a tertiary structure. In an attempt to find the structure-activity relationship of IAPPs, simulations of human, rat and other IAPP variants have been carried out previously. Replica exchange MD simulations with implicit solvent was used to show that non-aggregating porcine and rat IAPPs sample mostly helical conformations while aggregating human and cat IAPPs sample more β-hairpin structures^31^. In another MD study, the correlation between helicity of residues 7–16 and aggregation propensity of the peptide was observed — the more helical these residues are, the more aggregation-prone the corresponding peptide is Ref. 27. These two computational studies may appear to contradict with each other. However, our simulations showed a crossing of secondary structure contents between rIAPP and hIAPP as the temperature increases — at low temperatures rIAPP is relatively more helical, but at higher temperatures hIAPP can have more helical residues (Fig. S1). Therefore, the differences observed in previous simulations may result from different effective temperatures used corresponding simulations, as reported experimentally that the structural content of hIAPP depends on experimental conditions^41^,^44^.

Our DMD simulations also showed that hIAPP is stabilized upon dimerization. The dimer structure is driven by the interaction of amyloidogenic residues 22–29 of two monomers in a parallel orientation. The hIAPP monomer can also bind insulin monomers and dimers with the same residues forming hIAPP homodimer. Similarly, the residues of insulin monomer and dimer that bind hIAPP also participate in the formation of higher-molecular-weight insulin oligomers, i.e. dimer and hexamer, respectively. Our results are consistent with a number of previous structural studies, including the crystallography structure of IAPP fused to a maltose binding protein in a dimeric form, point mutation experiments, and computational studies^27^,^29^,^31^,^41^. Since insulin hexamer is usually insoluble and thus unavailable for binding, we did not model its interaction with hIAPP monomer. However, our results that hIAPP tends to bind the interface residues of insulin monomer and dimer in the 3D structure of insulin hexamer suggest that hIAPP-binding by insulin hexamer is weaker compared to monomer and dimer.

**Figure 5:**
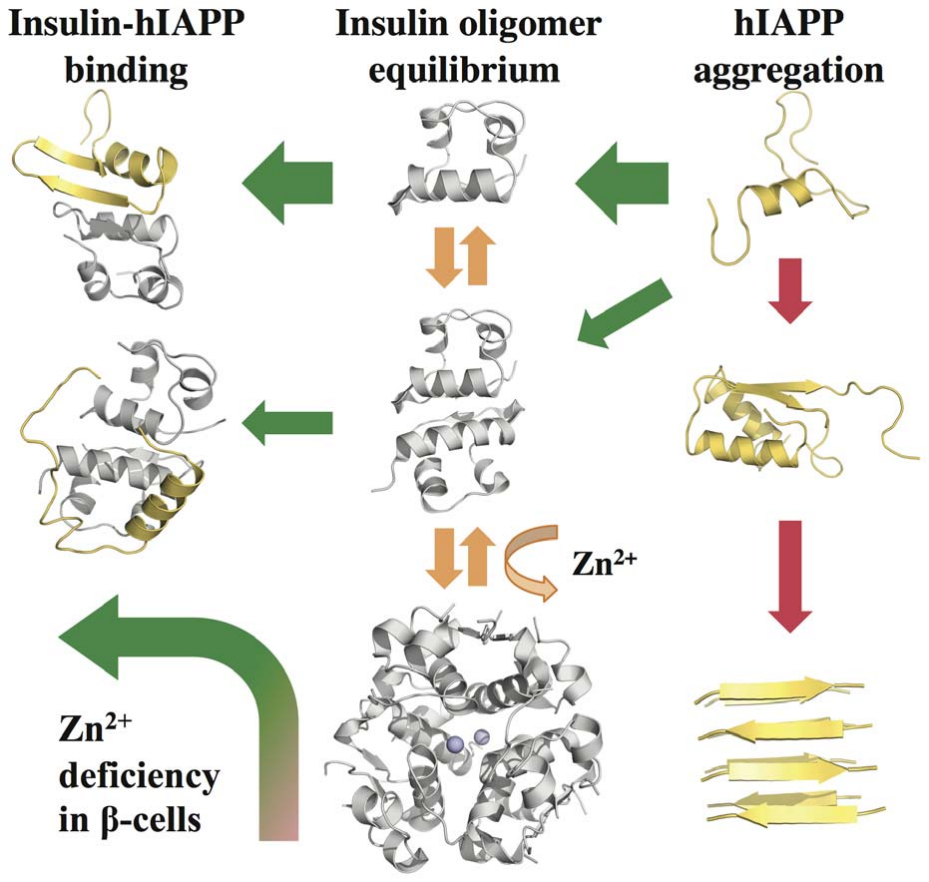
The relationship between hIAPP aggregation pathway and insulin oligomer equilibrium. hIAPPs (in yellow) form dimers first, and finally β-strand rich aggregates. Insulins (in gray) are at oligomer equilibrium between monomer, dimer and hexamer. Two zinc ions are required for the coordination of three dimers to form the hexamer, and thus the concentration of zinc ion governs the insulin oligomer equilibrium. Our DMD simulations suggest that hIAPP monomer preferentially binds to insulin monomer and dimer. Binding of hIAPP monomers by insulin monomers and dimers competes with hIAPP homodimer formation. With loss-of-function mutations in ZnT8, the deficiency of zinc ions shift the insulin oligomer equilibrium toward monomers and dimers, which sequester hIAPP monomers and inhibit hIAPP self-association and aggregation.

In β-cell granules, insulin outnumbers hIAPP by 1–2 orders of magnitude^16^. Because of a high concentration of zinc ions, insulin peptides are usually stored as insoluble, crystal-forming hexamers instead of soluble monomers and dimers^26^. The concentration of zinc ions in β-cell granule depends on the activity of ZnT8, which can be affected by mutations. Interestingly, the role of zinc ion in T2D is rather controversial at the population level since both anti-correlation^23^ and correlation^6^ are reported between ZnT8 activity and T2D risk. Our computational results are consistent with the latest GWAS study^6^ (Fig. 5). Specifically, the first step toward hIAPP aggregation is the homodimer formation^41^. hIAPP monomers can also bind to insulins, whose oligomer equilibrium is regulated by the zinc ion concentration. For the sub-population with loss-of-function mutations of ZnT8, the significantly low concentration of zinc ions shifts the insulin oligomer equilibrium towards soluble monomers and dimers. Binding of hIAPP monomers with insulin monomers and dimers competes with hIAPP dimerization. The availability of a high concentration of insulin monomers and dimers sequesters hIAPP monomers and inhibits the formation of hIAPP homodimers and subsequent amyloid aggregation, reducing T2D risk.

A major difference between the genetics association studies with “seemingly contradicting” correlations between T2D risk and ZnT8 activity is the extent of perturbation to ZnT8 transporter activity and thus the concentrations of zinc ions in β-cell granules. The Arg325Trp mutation only weakly perturbs the activity of ZnT8 and thus the base-line zinc ion concentrations, while loss-of-function mutations greatly reduce the concentration of zinc ions. We hypothesize that additional functions of zinc ion, such as direct binding of zinc with hIAPP^45^,^46^, might help explain the observed anti-correlation between Zn8T activity and T2D risk at high zinc concentrations. Therefore, a systems approach, which includes the molecular mechanism proposed in this work (Fig. 5) and additional inter-molecular interactions such as direct zinc-insulin binding, is necessary to reconcile various complex effects and sometimes paradoxical observations in the field of T2D research, to uncover the disease mechanism, and design therapeutics.

## Methods

### System setup and simulation details

The simulated molecular systems included hIAPP monomer, rIAPP monomer, hIAPP homodimer, human insulin monomer (consisting of chains A and B), hIAPP dimer with insulin monomer and hIAPP monomer with insulin dimer. The protein coordinates were obtained from the protein data bank, PDB codes 2L86^38^ for hIAPP, 2KJ7^40^ for rIAPP, and 1TRZ^47^ for insulin.

Simulations were performed using discrete molecular dynamics (DMD) algorithm^34^,^35^. Briefly, DMD uses step-function potentials to model the interactions between atoms. During the course of simulation atoms interact via series of collisions between which the velocities remain constant. At each collision, the velocities are updated following the conservation of energy, momentum and angular momentum. We used the united-atom representation for proteins. The interaction potential includes van der Waals, solvation, environment-dependent hydrogen bonding and electrostatic interactions in addition to the bonded terms. The solvation energy was modeled using the Lazaridis-Karplus implicit solvation^48^. The hydrogen bond interaction was modeled using a reaction-like algorithm^49^. We also included electrostatic interactions between charges, including the basic and acidic residues in proteins^50^. The Debye-Hückel approximation, with Debye length setting to 10 Å, was used to model the screened charge-charge interactions. We used an interaction range of 30 Å for the electrostatic interactions, where the screened potential approaches zero.

We performed both constant temperature and replica exchange DMD (REXDMD) simulations. The replica exchange simulations have higher sampling efficiency compared to constant temperature simulations and hence are suitable for calculating equilibrium properties. However, for protein-protein interactions, the vast available conformational space makes it challenging for even replica exchange simulations to reach the association/dissociation equilibrium. Starting from the bound state of a molecular complex, replica exchange simulations can be used to sample the *dissociation* dynamics and the corresponding dissociation temperature can be calculated to estimate the binding strength of the complex. In order to capture the *association* dynamics, we performed multiple independent constant temperature simulations with different initial starting structures, including inter-molecular distances and orientations. Although we do not expect each simulation to reach the association/dissociation equilibrium, by averaging over all these independent simulations at a given temperature we expect to obtain a better estimation of association-related quantities, such as the hIAPP-binding frequency of each insulin residue, with a higher statistical significance.

### Constant temperature simulations

In constant temperature simulations, we performed ten independent DMD runs with randomly chosen initial conditions (velocity of atoms; relative orientations of monomers in the case of multiple proteins). For the largest system of insulin dimer–hIAPP, 20 independent DMD simulations were performed to ensure sufficient sampling. Each simulation lasted two million DMD time units or 100 ns (each time unit corresponds to 50 fs). We found that the calculated quantities such as secondary structure contents and contact numbers often rapidly reached plateau (e.g. typical DMD simulations in Fig. S5). Therefore, by starting from different initial structures, running long simulations, and averaging with multiple independent simulations, we expected to reduce possible simulation biases, increase sampling efficiency, and improve the convergence of calculations. The temperatures in DMD simulations were maintained using the Andersen thermostat^51^. The scaling factor for converting these reduced temperature units to the corresponding values in Kelvin is approximately 5.03 × 10^2^.

### Replica exchange simulations

In REXDMD simulations, multiple simulations or replicas of the same molecular system were performed in parallel at different temperatures. The replicas periodically reshuffle simulation temperatures according to the replica exchange algorithm^52^ for enhanced sampling efficiency. Eight replicas with temperatures evenly sampled within the range from 0.5 to 0.675 (reduced units) were simulated. The temperatures were chosen to ensure that the acceptance ratio for exchange is between 0.3 and 0.7. The simulations were carried out for 2 million DMD time units as in the constant temperature DMD simulations. The temperature dependence of thermodynamic quantities are calculated using the weighted histogram analysis method (WHAM)^39^.

### Clustering

First, the trajectories from independent simulations were concatenated and low energy frames were selected from this combined trajectory. The cutoff energy used for determining low energy frames was determined for each system such that about one-fourth of the frames are selected. Similar structures with consecutive frames were then filtered out using a sliding window (~3000–4000 DMD time units). The resulting configurations were clustered into fifty clusters using the hierarchical clustering algorithm, OC^42^. Within each cluster, the conformation that has the minimum RMSD to all other structures was chosen as the centroid.

### Contact frequency map

Two residues were defined to be in contact if the distance between any of the atom pairs between them was less than 5Å. The contact frequency of any pair of residues was the ratio of contact time to the total time, calculated over the later half of the simulations.

## Author Contributions

P.N.G. and F.D. conceived the research design. P.N.G. performed the computational simulations and data analysis. P.N.G. and F.D. wrote the manuscript.

## Supplementary Material

Supplementary Information Supplementary Figures
