# Supplementary material for: Inhibition of IAPP aggregation by insulin depends on the insulin oligomeric state regulated by zinc ion concentration
Source: Sci Rep. 2015 Feb 4;5:8240. doi: 10.1038/srep08240 (PMC4316164; doi:10.1038/srep08240)
Supplement: Supplementary Information — Supplementary Figures [file srep08240-s1.pdf]

Supporting Information

Inhibition of IAPP aggregation by insulin depends on the insulin oligomeric state regulated by zinc ion concentration

Praveen Nedumpully-Govindan[1] and Feng Ding[1]

[1]Department of Physics and Astronomy, Clemson University, Clemson, SC 29634, USA
*Corresponding Author. E-mail: fding@clemson.edu

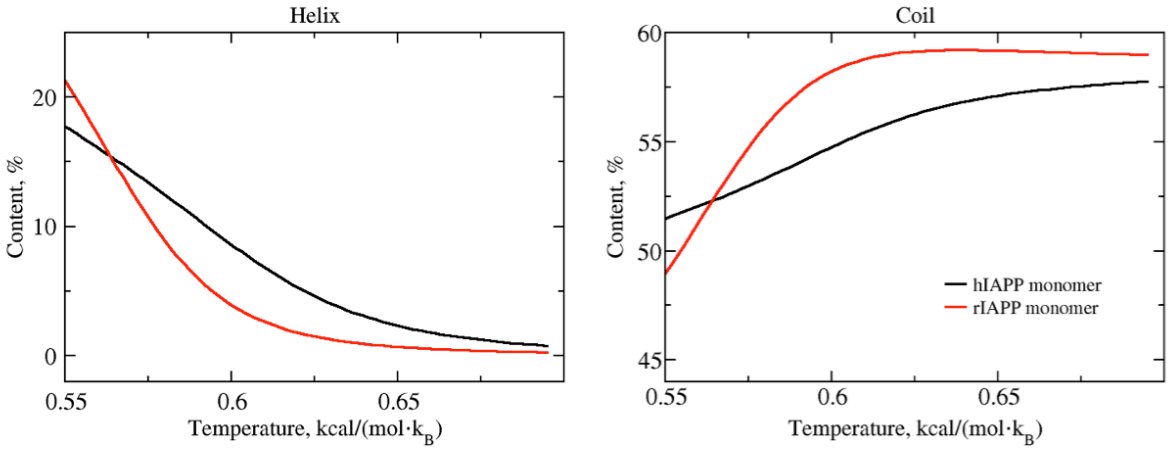

Figure S1. The temperature dependence of secondary structure contents of hIAPP and rIAPP peptide monomers. As the temperature increases, the helical content of the peptides decreases while the coil content increases. This change is slow and steady for hIAPP, but rIAPP curves have a sharper transition point. The different behaviors of the two IAPP variants support the observation from the specific heat plot (Fig. 1) that hIAPP is structurally disordered whereas rat peptide features a well-defined tertiary fold.

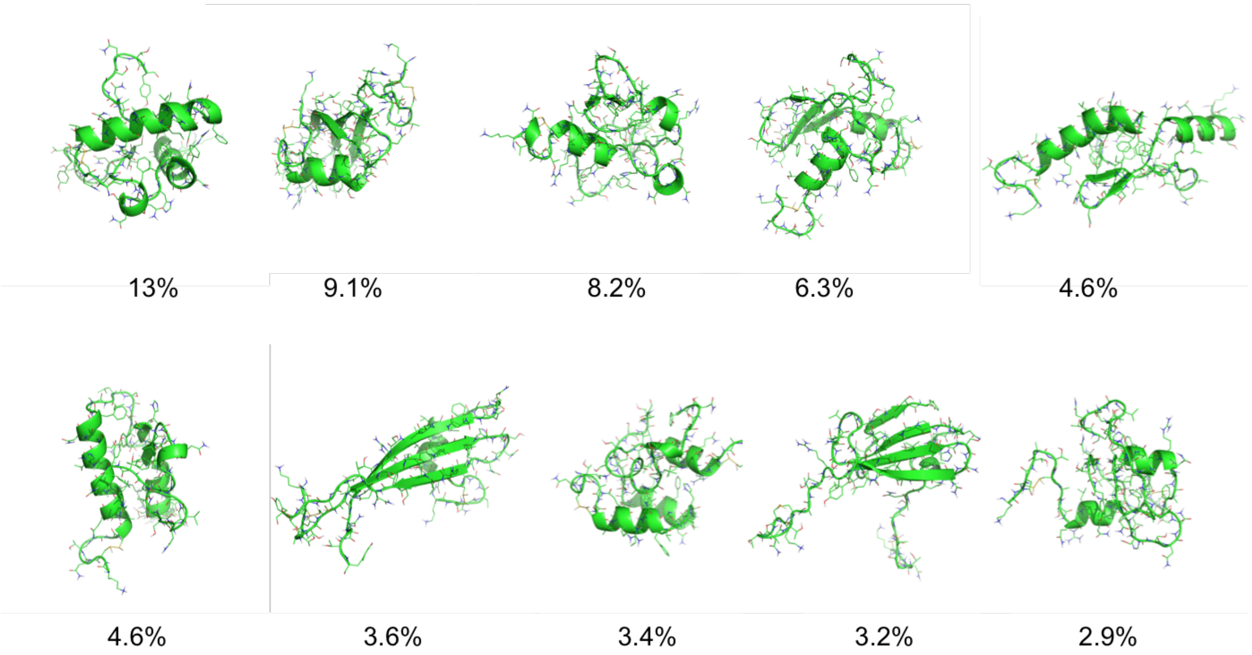

Figure S2. hIAPP dimer centroid structures. The low energy conformations obtained from hIAPP dimer simulations are clustered according to their mutual RMSDs. The centroid representatives of ten largest clusters are shown in cartoon representation. The population of each cluster is also shown in terms of the percentage of total number of conformations.

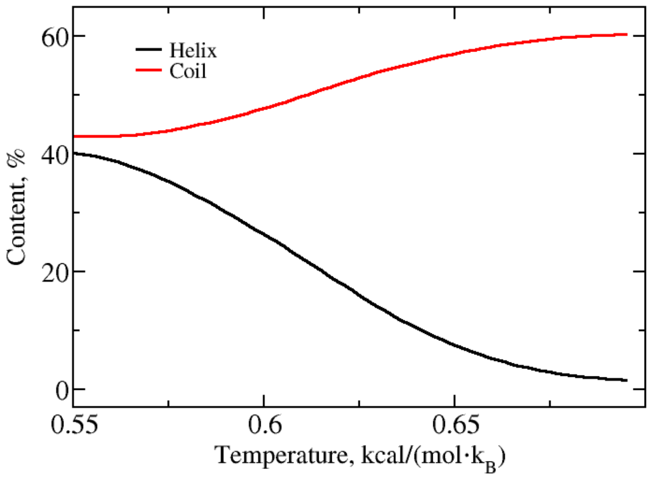

Figure S3. Temperature dependence of hIAPP secondary structure content when it is bound to insulin. At low temperatures, the helical content is higher in comparison to hIAPP monomer (Fig. S1), but it drops quickly as the temperature increases. A similar but opposite trend is observed for the coil content.

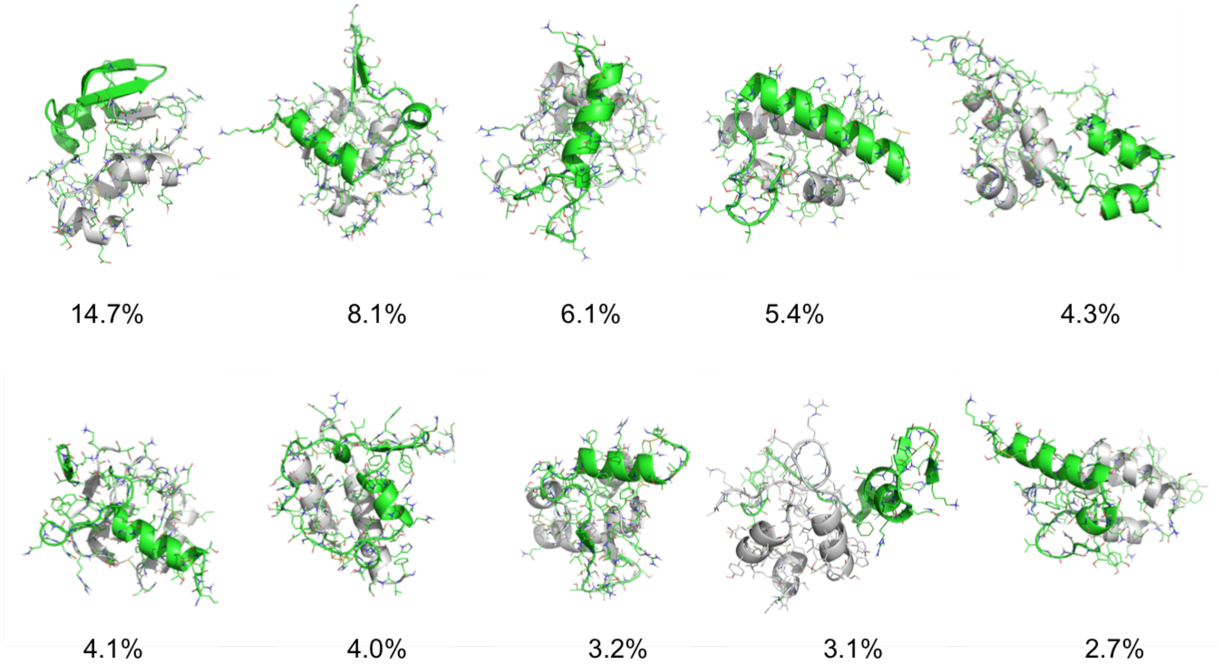

Figure S4. hIAPP-insulin dimer centroid structures. The low energy conformations obtained from insulin–hIAPP dimer simulations are clustered according to their mutual RMSDs. The centroid representatives of ten largest clusters are shown in cartoon representation. The population of each cluster is also shown in terms of percentage of total number of conformations. The insulin is shown in gray and hIAPP in green.

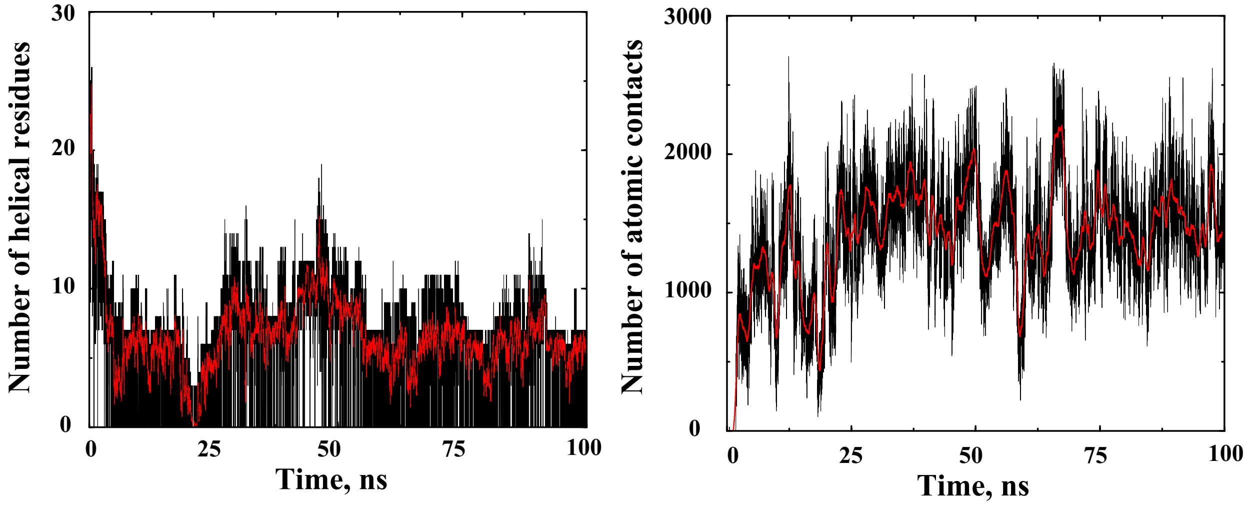

Figure S5. The time-dependence of estimated quantities in DMD simulations. The time-dependence of the number of helical residues is shown from a hIAPP-hIAPP dimer simulation (left), and the number of atomic contacts between hIAPP and insulin is presented from an insulin monomer-hIAPP simulation (right). These quantities rapidly reach their mean values, and fluctuate afterwards. These simulations are arbitrarily chosen without any average. The red lines represent the running average values using windows of 20 (helical residue number) or 50 (contact number) snapshots.
